# Effectiveness of Intermittent Preventive Treatment in Pregnancy with Sulphadoxine-Pyrimethamine against Submicroscopic *falciparum* Malaria in Central Region, Ghana

**DOI:** 10.1155/2015/959427

**Published:** 2015-09-13

**Authors:** Ekene K. Nwaefuna, Richmond Afoakwah, Verner N. Orish, Alexander Egyir-Yawson, Johnson N. Boampong

**Affiliations:** ^1^Vector Genetics Laboratory, Biotechnology and Nuclear Agriculture Research Institute, Ghana Atomic Energy Commission, Accra, Ghana; ^2^Department of Biomedical and Forensic Sciences, University of Cape Coast, Cape Coast, Ghana; ^3^Department of Internal Medicine, Effia-Nkwanta Regional Hospital Sekondi-Takoradi, Sekondi, Western Region, Ghana

## Abstract

Malaria infections undetectable by microscopy but detectable by Polymerase Chain Reaction (PCR) (submicroscopic malaria) are common in endemic areas like Ghana. Submicroscopic malaria has been linked with severe pregnancy outcomes as well as contributing to malaria transmission. In this cross-sectional study 872 consenting pregnant women (gestation ≥ 20 weeks) were recruited from 8 hospitals in Central Region, Ghana, between July and December 2009. Malaria infection was detected by microscopy and PCR. Haemoglobin was measured and anaemia was defined as haemoglobin lower than 11 g/dL. Majority of the women, 555 (63.6%), were Intermittent Preventive Treatment in Pregnancy with Sulphadoxine-Pyrimethamine (IPTp-SP) users while 234 (36.4%) were nonusers. The prevalence of malaria by microscopy was 20.9% (182/872) and 9.7% (67/688) of microscopy negative women had submicroscopic malaria. IPTp-SP usage significantly (odds ratio = 0.13, 95% confidence interval = 0.07–0.23, *p* = 0.005) reduced the prevalence of submicroscopic malaria as more nonusers (51/234) than users (16/454) were PCR positive. After controlling for other variables the effect of IPTp-SP remained statistically significant (odds ratio = 0.11, 95% confidence interval = 0.02–0.22, *p* = 0.006). These results suggest that Intermittent Preventive Treatment with Sulphadoxine-Pyrimethamine is useful in the reduction of submicroscopic malaria in pregnancy.

## 1. Introduction

Malaria accounts for the highest cause of mortality in Africa with 90% of the global malaria deaths occurring on this continent. It constitutes 9% of the disease burden to the people of Africa and is responsible for 25% of deaths below the age of five years [1]. Susceptibility to malaria is increased by up to 3-fold during pregnancy, thereby making pregnant women one of the most vulnerable groups [2].

Submicroscopic malaria, defined as plasmodium infection below the threshold detectable by microscopy but detectable by Polymerase Chain Reaction (PCR), has received lots of attention due to its role in disease transmission [3]. Due to the sequestration of* Plasmodium falciparum *infected red blood cells in the placenta of pregnant women, malaria diagnosisfrom peripheral blood has been inadequate. However PCR diagnosis is effective in detecting not only parasitaemia below the threshold of microscopy but also placental infections [4]. PCR has been reported to be more sensitive in the detection of malaria during pregnancy than the microscopy of placenta impression smears; 65% of women negative for malaria by microscopy were positive by PCR [5].

In areas of stable malaria transmission in Africa, the WHO recommends a package of Intermittent Preventive Treatment in Pregnancy with Sulphadoxine-Pyrimethamine (IPTp-SP) and use of Insecticide Treated Nets. Nonetheless, achievement of high coverage of these preventive interventions among pregnant women remains elusive for many countries in Sub-Saharan Africa [1]. In Ghana, the WHO recommendations were adopted and finally implemented in 2005; however coverage remains generally low. Nonetheless, several studies have continued to report its effectiveness in prevention of pregnancy associated malaria [6–8]. It, however, becomes necessary to continue to access its usefulness in varying dynamics of pregnancy associated malaria. In this study, we investigated the effectiveness of IPTp-SP against submicroscopic malaria among pregnant women in Central Region, Ghana, barring all constraints associated with the administration of this intervention.

## 2. Materials and Methods

### 2.1. Study Area and Population

The study area has been described elsewhere [9], but briefly the study was carried out in Central Region, Ghana. Central Region (5°30′0′′N, 1°0′0′′W) occupies an area of 9,826 square kilometers, which is about 6.6% of the land area of Ghana. It has an estimated population of 1,805,488 and an annual population growth rate of 2.1% with 17 administrative districts [10]. In 2008, the region registered 86,971 pregnant women thus recording 115.5% antenatal care coverage and 56.3% supervised delivery [11].

### 2.2. Ethical Clearance

Ethical approval was sought from the Ghana Health Service Ethical Review Committee (ID number: GHS – ERC – 13/7/09). All activities including sample collection, processing, and analysis were carried out as required by the committee. The Central Regional Health Directorate also approved the protocols used in this study.

### 2.3. Study Design

This research was a cross-sectional study. The sample sites (hospitals) were purposefully selected while the participants randomly selected. The region was divided into two belts and four hospitals were selected from each belt: the Coastal Belt (15 km inland) (Moree Health Centre, Kasoa Health Centre, Elmina Health Centre, and Cape Coast Metropolitan Hospital) and the Forest Belt (Swedru Hospital, Our Lady of Grace Hospital Asikuma, Saint Francis Xavier Hospital, Assin Foso, and Dunkwa-on-Offin District Hospital). The study was designed to evaluate the effectiveness of IPTp-SP in the normal state of its implementation barring all challenges and obstacles as a result of health system problems. However, only hospitals with functional ANC clinics where the IPTp-SP policy is implemented were included in the study.

### 2.4. Sample Size Determination

Using the method described by Fisher a sample size of seven hundred and ten (710) was required to represent the population of pregnant women in Central Region based on the following assumptions: standard normal deviation of 1.96 corresponding to 95% confidence interval, 29% prevalence of malaria in pregnancy, and 0.05 degrees of accuracy [12]. The total number of pregnant women reporting to each health facility yearly was made as a fraction of the total number of pregnant women seen in all these health facilities annually. The proportion for each facility was used to determine the number of pregnant women sampled from that facility. However eight hundred and seventy-two (872) samples were collected.

### 2.5. Selection Criteria

The pregnant women were recruited from antenatal care units of the hospitals after written informed consent was obtained according to the Ethical Review Committee of the Ghana Health Service. Only pregnant women ≥ 20 weeks (second or third trimester) were recruited. Pregnant women with complications such as sepsis and hemorrhage were excluded.

### 2.6. Data Collection

Eight hundred and seventy-two [872] pregnant women participated in the study. Some health and obstetric pieces of information such as gestation, gravidity, Glucose-6-Phosphate Dehydrogenase (G6PD) status, and SP intake were obtained from the Antenatal Clinic (ANC) booklets of participants; other information was collected using a structured questionnaire administered by trained research assistants. The assistants were recruited and trained to explain the questionnaire in their own local languages. Three milliliters (3 mL) of venous blood was collected from the median cubital or cephalic veins of the arm of pregnant women into labeled vacutainers, containing Ethelene Diamine Tetra Acetic Acid (EDTA). One hundred microliters (100 *μ*L) of the blood was spotted on Whatman No. 1 filter papers, air dried and placed in labeled plastic envelopes, and then stored at −20°C until usage. Thick blood films were prepared on labeled microscope slides, Giemsa-stained and observed by a trained and licensed senior biomedical scientist and a principal biomedical scientist for malaria diagnosis. A sample was labelled positive if one or more parasites are present and negative if none is seen after observing 100 high power fields. Haemoglobin (Hb) level was determined using the SYSMEX KX – 21 haematology analyser (Sysmex Corporation, Kobe, Japan) and the numerical values were recorded. The sodium metabisulphite method was used to determine sickling status as described by Cheesbrough [13].

### 2.7. Molecular Analysis

**Figure 1:**
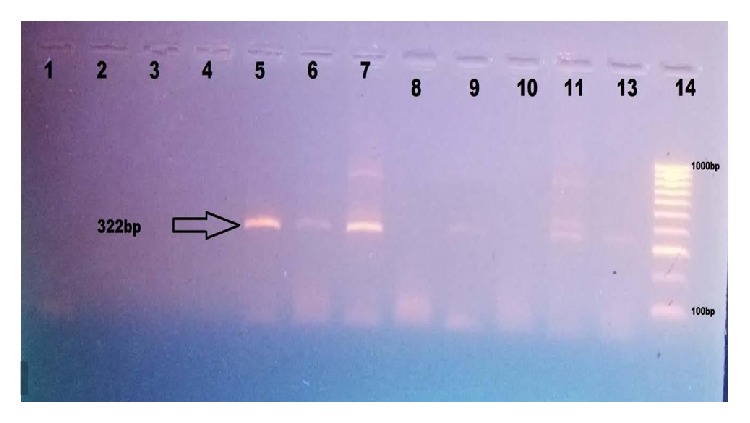
Gel photograph of the 2nd amplification, showing the 322 bp product. Lane 1 = negative control, lane 5 = positive control, and lanes 2–4 and 6–13 = participants' samples.

*Plasmodium falciparum *DNA was extracted from blood spots on filter paper using the CHELEX Method of extraction as described by Bereczky et al. [14]. Extracted DNA samples were stored at −20°C until usage. Of the 690 participants who were malaria negative by microscopy and included in the molecular analysis, only 688 were successful. A nested Polymerase Chain Reaction (PCR) was carried out following the method described by Beck and Ley [15] with slight modifications. The primary PCR amplified a 642 bp* Plasmodium falciparum* dihydrofolate reductase (*pfdhfr*) gene. The reaction was set up in a 96-well plate to a final volume of 25 *μ*L per well containing 1x buffer, 1.5 mM MgCl_2_, 200 *μ*M dNTP mix, and 0.25 *μ*M of each primer (M_1_ = 5′ TTT ATG ATG GAA CAA GTC TGC 3′, M_5_ = 5′ AGT ATA TAC ATC GCT AAC AGA 3′). One denaturing step at 94°C for 3 minutes was followed by 40 amplification cycles. Each cycle constituted a denaturation step at 94°C for 1 minute, an annealing step at 50°C for 2 minutes, and an extension step at 72°C for 2 minutes. This was followed by a final extension step 72°C for 10 minutes. This cycler condition lasted for 4 hours 30 minutes. Five microlitres of the primary PCR product was used for the second amplification using the following primers: (F′ = 5 GAA ATG ATG GAA CAA GTC TGC GAC GTT 3′) and (2F = 5′ TTA ATT TCC CAA GTA AAA CTA TTA GAG CTT3′) corresponding to codon 59–108 of the* pfdhfr* gene. The reaction was set up in a 96-well plate to a final volume of 25 *μ*L per well containing 1x buffer, 1.5 mM MgCl_2_, 200 *μ*M dNTP mix, and 0.25 *μ*M of each primer. One denaturing step at 94°C for 2 minutes was followed by 40 amplification cycles. Each cycle constituted a denaturation step at 94°C for 1 minute, an annealing step at 45°C for 1 minute, and an extension step at 72°C for 2 minutes. This was followed by a final extension step 72°C for 10 minutes. This cycler condition lasted for 3 hours 30 minutes. Eight microlitres (8 *μ*L) of each nested PCR product was separated on 2% agarose gel stained with ethidium bromide. The gels were then photographed (Figure 1) under UV light. Any sample with a nested PCR product that showed a band equivalent to the 322 bp mark on the molecular weight marker was labeled as positive for malaria, specifically* Plasmodium falciparum *infection.

### 2.8. Definitions

IPTp-SP users are pregnant women who have taken at least one dose of SP and no other malaria prophylaxis while nonusers have not taken SP during the present pregnancy at the time of the study. Gravidity was grouped as Primigravidae (1st pregnancy), Secundigravida (2nd pregnancy), and multigravidae (3rd or more pregnancies). A malaria positive blood smear was regarded as microscopy positive. A microscopy negative but PCR positive sample was regarded as submicroscopic malaria. A participant was anaemic if Hb < 11.0 g/dL.

### 2.9. Statistical Analysis

All data was entered into SPSS statistical software version 17.0 for Windows and analyzed. Frequencies and Pearson's chi-square were used to compare relationships between variables. To determine the independent effect of IPTp-SP on submicroscopic malaria, logistic regression modeling was applied. Possible confounding factors such as gestation, occupation, gravidity, G6PD, and sickling status were controlled. Odds ratio (OR) and confidence interval (CI) were calculated by using logistic regression in multivariate analyses. In the logistic regression analysis, all variables were treated as categorical except gestation and gravidity that were treated as categorical. For all statistical tests, *p* < 0.05 was considered significant.

## 3. Results

### 3.1. General Characteristics of Participants

A total of 872 pregnant women consented to participate in the study. The mean age of the participants was 32.9 years ranging from 17 to 46 years. When grouped by IPTp-SP usage, there was no significant difference (*p* = 0.33) in the mean age for users 33.4 years range (20–46) and nonusers 32.1 (17–44). Women using IPTp-SP were significantly more educated (*p* = 0.011) and better employed (*p* = 0.001) than nonusers (Table 1).

### 3.2. Health and Obstetric Information of Participants

There was a significant difference between the IPTp-SP users and nonusers based on trimester (*p* = 0.001) and gravidity (*p* = 0.023) as more women with pregnancy experience were users than nonusers. There was no association between the two groups in terms of G6PD (*p* = 0.719) and sickling status (*p* = 0.123) (Table 2).

### 3.3. Submicroscopic Malaria in Pregnancy

The prevalence of submicroscopic malaria was not significantly affected by the gestation (*p* = 0.575) and gravidity (*p* = 0.665). More than half of the women with submicroscopic malaria were anaemic. Submicroscopic malaria significantly resulted in anaemia (*p* = 0.003) (Table 3).

Increasing dosage of SP had a significant (*p* = 0.018) preventive effect on submicroscopic malaria. The prevalence of malaria by microscopy was 20.9% (182/872) and by PCR (submicroscopic) was 9.7% (67/688) but when submicroscopic infection was added it shot up to 28.6% (249/872). IPTp-SP usage significantly reduced submicroscopic* falciparum* infection (OR = 0.13, 95% CI = 0.07–0.23, *p* = 0.005) (Table 4).

From the logistic regression, after controlling for possible confounders, the effect of IPTp-SP and gestation on submicroscopic malaria remained statistically significant (OR = 0.11, 95% CI = 0.02–0.22, *p* = 0.006; OR = 0.28, 95% CI = 0.18–0.42, *p* = 0.001) (Table 5).

## 4. Discussion

Eight hundred and seventy-two (872) consenting pregnant women participated in the study. All the participants were eligible to use SP but only 555/872 (63.6%) had used IPTp. This may be because the women report late for antenatal care or they do not visit regularly. Though data on ANC attendance was not collected in the present study, late first ANC clinic enrolments and fewer visits have been reported to limit IPTp-SP usage [16]. IPTp-SP users were not significantly (*p* = 0.33) older than nonusers but weresignificantly more educated and better employed than nonusers. This should buttress the importance of education in health care; as such health education schemes for pregnant women should be intensified in rural areas. The social status of Ghanaians has often been linked to health care as people with stable income have been shown to exhibit better health seeking behavior [17].

More women with pregnancy experience were IPTp users; this might be because the women were already conversant with the routine procedures at the ANC. Thus, they reported early and regularly. More third trimester participants were IPTp users as there was a significant association between gestation and IPTp usage. This is not surprising because at the third trimester pregnant women are expected to have had several ANC visits during which the IPTp intervention should have been administered. G6PD deficiency and sickling status did not vary significantly between the two groups.

The prevalence of malaria by microscopy was 20.9% (182/872) while that of submicroscopic malaria was 9.7% (67/688). Submicroscopic malaria in pregnancy may be as a result of parasitaemia below the threshold of microscopy or the sequestration of infected red blood cells in the placenta [18]. These results are different from findings of a similar study in Kumasi, Ghana, where prevalence by microscopy was 51%, PCR was 49%, and both were 63% [19]. Despite the fact that both studies were carried out in different geographical areas of Ghana, the Kumasi study was conducted prior to the implementation of IPTp-SP in Ghana; thus the difference in prevalence may be as a result of the implementation of malaria control measures.

A number of studies have suggested that malaria infection of any parasite density may have a harmful effect on pregnant women and their developing fetuses [20, 21]. Submicroscopic malaria has been highly implicated for its contribution to malariatransmission as well as complicating control programmes and treatment [22]. In a recent study carried out in the Sudan, pregnant women with submicroscopic malaria had a higher risk of having low birth weight babies [23]. Since malaria elimination programs have been directed towards mass screening and treatment of asymptomatic individuals, further research should strive to elucidate the degree to which submicroscopic malaria contributes to the infectious reservoir and, in turn, what diagnostic detection threshold is needed to effectively interrupt transmission [3].

The prevalence of submicroscopic malaria did not differ significantly by gestation and gravidity (*p* = 0.575 and 0.665), respectively. This is contrary to an earlier study that linked higher submicroscopic infections to primigravidae [19]. However after controlling for possible confounding factors the prevalence of submicroscopic malaria became significant by gestational age (*p* = 0.001). More than half (52%) of the participants with submicroscopic malaria were anaemic (Hb < 11.0 g/dL). Anaemia in pregnancy is however multifactorial and can be caused by a number of reasons including poor nutrition, intestinal parasites, and socioeconomic status which have not been measured in this study. Nonetheless, similar studies have reported the contribution of malaria to anaemia in pregnancy [6, 24]. This is indicative of the effect of submicroscopic* P*.* falciparum* infection in pregnancy. These submicroscopic infections may be as a result of prevailing placenta infection, because diagnosis of placenta malaria from peripheral blood by PCR has been reported to approach 100% accuracy [25].

Due to the profound consequences of submicroscopic malaria, it is very important that the IPTp intervention should not only be effective in clearing parasite from the peripheral blood but also prevent submicroscopic and placental infections. Usage of IPTp significantly reduces the presence of malaria infection both at microscopic and submicroscopic levels (*p* = 0.002, *p* = 0.005, resp.). Studies have reported the protective efficacy of Sulphadoxine-Pyrimethamine when used as malaria prophylaxis during pregnancy. We further indicate here that its efficacy extends beyond the threshold of microscopic to submicroscopic infections. Recent studies have reported treatment failures as well as rise in the prevalence of molecular markers of SP resistance. In Ghana, for example, prevalence of molecular markers of SP resistance was 73%; nevertheless SP remains effective in preventing Ghanaian women from malaria [6–8]. It becomes important for further research to be carried out to clarify the relationships between drug resistance, treatment failure, and molecular markers of SP resistance. The prevalence of submicroscopic malaria reduced significantly (*p* = 0.018) with increasing dosage of SP. One clinical trial in Malawi also underscored the increased efficacy of SP with increasing doses among delivering women [26].

The major limitation of this study is the absence of data on pregnancy outcomes such as placental malaria, congenital and perinatal mortality, and birth weight. This was due to the cross-sectional nature of the study and did not allow for participants to be followed till delivery. However, reports have been made on the useful effect of IPTp-SP in the reduction of low birth weight (LBW), neonatal mortality (NM), and placental malaria (PM) [8, 24, 25, 27]. Another limiting factor may be the general health systems barriers, which include staff motivation, lack of skilled personnel, and availability of medications, capable of hindering the success of health care policies in Sub-Saharan Africa [28, 29]. However, only hospitals with vibrant ANC clinics were selected for this study: an attempt to limit the effect of this limitation.

Intermittent Preventive Treatment in Pregnancy with Sulphadoxine-Pyrimethamine continues to be a useful regimen for the prevention of pregnancy associated malaria and submicroscopic malaria in pregnancy.

## 5. Conclusion

IPTp-SP is useful in the prevention of pregnancy associated malaria in Ghana.

## Figures and Tables

**Table 1:** Background characteristics of participating women.

Variables	IPIp-SP	No IPTp-SP	*p*
	(*n* = 555)	(*n* = 317)	
Mean age ± SD	33.4 ± 4.9	32.1 ± 5.1	0.33
Range	20–46	17–44	
Educational level			0.011
None	93 (16.8%)	59 (18.6%)	
Primary	100 (18.0%)	39 (12.4%)	
Secondary	336 (60.5%)	189 (59.5%)	
Tertiary	26 (4.6%)	30 (9.5%)	
Occupation			0.001
Unemployed	93 (16.8%)	46 (14.7%)	
Unskilled	299 (54.0%)	145 (46.0%)	
Para-profession	145 (26.1%)	95 (30.0%)	
White collar	10 (1.8%)	9 (2.8%)	
Student	8 (1.4%)	22 (6.9%)	

**Table 2:** Health and obstetric information of participants.

Variables	IPIp-SP	No IPTp-SP	*p*
	(*n* = 555)	(*n* = 317)	
Trimester			0.0001
Second	397 (71.5%)	285 (89.9%)	
Third	158 (28.5%)	32 (10.1%)	
Gravidity			0.023
Primigravidae	146 (26.3%)	111 (35.0%)	
Secundigravida	167 (30.1%)	88 (27.8%)	
Multigravidae	242 (43.6%)	118 (37.2%)	
G6PD			0.719
No defect	551 (99.3%)	314 (99.1%)	
Partial defect	4 (0.07%)	3 (0.09%)	
Sickling status			0.123
Positive	61 (11.0%)	17 (5.4%)	
Negative	494 (89.0%)	300 (94.6%)	

**Table 3:** Submicroscopic malaria dynamics among pregnant women in Ghana.

Variables	Submicroscopic malaria		*p*
	Positive (*n* = 67)	Negative (*n* = 621)	
Trimester			0.575
Second	51 (76.1%)	491 (79.1%)	
Third	16 (23.9%)	130 (20.9%)	
Gravidity			0.665
Primigravidae	16 (23.9%)	172 (27.7%)	
Secundigravida	23 (34.3%)	183 (29.5%)	
Multigravidae	28 (41.8%)	266 (42.8%)	
Anaemia			0.003
Anaemic	35 (52.2%)	210 (33.8%)	
Normal	32 (47.8%)	411 (66.2%)	
SP dosage			0.018
None	16 (23.9%)	218 (35.1%)	
Once	36 (53.7%)	293 (47.2%)	
Twice	14 (20.9%)	86 (13.8%)	
Thrice	1 (1.5%)	24 (3.9%)	

**(a):** 

Variable	IPIp-SP	No IPTp-SP	Odds ratio	95% CI	*p*
	(*n* = 454)	(*n* = 234)			
Submicroscopic			0.13	0.07–0.23	0.005
Positive	16 (3.5%)	51 (21.8%)			
Negative	438 (96.5%)	183 (79.2%)			

**(b):** 

Variables	IPIp-SP	No IPTp-SP	Odds ratio	95% CI	*p*
	(*n* = 555)	(*n* = 317)			
Microscopic			0.08	0.05–0.13	0.002
Positive	38 (6.8%)	144 (45.4%)			
Negative	517 (93.2%)	173 (54.6%)			
Microscopic + submicroscopic			0.06	0.04–0.09	0.001
Positive	54 (9.7%)	195 (61.5%)			
Negative	551 (90.3%)	139 (39.5%)			

**Table 5:** Logistic regression analyses of confounders of submicroscopic malaria infection among pregnant women from selected health facilities in Central Region, Ghana.

Variable	Odds ratio	95% CI	*p*
Education	1.02	0.84–1.09	0.721
Gravidity	0.74	0.31–1.73	0.092
Occupation	1.55	0.42–4.40	0.041
Gestation	0.28	0.18–0.42	0.001
Sickle cell positive	2.18	1.24–3.80	0.061
IPTp-SP	0.11	0.02–0.22	0.006
G6PD partial defect	1.75	0.44–7.06	0.428

## References

[1] WHO (2009). *The World Malaria Report*.

[2] Sappenfield E., Jamieson D. J., Kourtis A. P. (2013). Pregnancy and susceptibility to infectious diseases. *Infectious Diseases in Obstetrics and Gynecology*.

[3] Lin J. T., Saunders D. L., Meshnick S. R. (2014). The role of submicroscopic parasitemia in malaria transmission: what is the evidence?. *Trends in Parasitology*.

[4] Mockenhaupt F. P., Bedu-Addo G., von Gaertner C. (2006). Detection and clinical manifestation of placental malaria in southern Ghana. *Malaria Journal*.

[5] Omer S., Khalil E., Ali H., Sharief A. (2011). Submicroscopic and multiple plasmodium falciparum infections in pregnant Sudanese women. *North American Journal of Medical Sciences*.

[6] Wilson N. O., Ceesay F. K., Obed S. A. (2011). Intermittent preventive treatment with sulfadoxine-pyrimethamine against malaria and anemia in pregnant women. *American Journal of Tropical Medicine and Hygiene*.

[7] Asa O. O., Onayade A. A., Fatusi A. O., Ijadunola K. T., Abiona T. C. (2008). Efficacy of intermittent preventive treatment of malaria with sulphadoxine-pyrimethamine in preventing Anaemia in pregnancy among Nigerian women. *Maternal and Child Health Journal*.

[8] Gies S., Coulibaly S. O., Ouattara F. T., D'Alessandro U. (2009). Individual efficacy of intermittent preventive treatment with sulfadoxine-pyrimethamine in primi- and secundigravidae in rural Burkina Faso: impact on parasitaemia, anaemia and birth weight. *Tropical Medicine and International Health*.

[9] Afoakwah R., Boampong J. N., Egyir-Yawson A., Nwaefuna E. K., Verner O. N., Asare K. K. (2014). High prevalence of *PfCRT* K76T mutation in *Plasmodium falciparum* isolates in Ghana. *Acta Tropica*.

[10] http://www.wikipedia.org/wiki/Central_Region.

[11] http://www.ghanahealthservice.org/ghs-region.php?ghs&ghsrid=5.

[12] Zhang H., Stern H. (2009). Sample size calculation for finding unseen species. *Bayesian Analysis*.

[13] Cheesbrough M. (2005). *District Laboratory Practice in Tropical Countries (Part 1)*.

[14] Bereczky S., Mårtensson A., Gil J. P., Färnert A. (2005). Short report: rapid DNA extraction from archive blood spots on filter paper for genotyping of *Plasmodium falciparum*. *The American Journal of Tropical Medicine and Hygiene*.

[15] Beck H., Ley S. C., Mell K., Ljungstrom I., Perlmann H., Scherf A., Wahlgren M. (2008). Monitoring of malaria drug resistance associated SNPs in *Plasmodium falciparum* on microarray. *Methods in Malaria Research*.

[16] Anchang-Kimbi J. K., Achidi E. A., Apinjoh T. O. (2014). Antenatal care visit attendance, intermittent preventive treatment during pregnancy (IPTp) and malaria parasitaemia at delivery. *Malaria Journal*.

[17] Danso-Appiah A., Stolk W. A., Bosompem K. M. (2010). Health seeking behaviour and utilization of health facilities for schistosomiasis-related symptoms in Ghana. *PLoS Neglected Tropical Diseases*.

[18] Okell L. C., Ghani A. C., Lyons E., Drakeley C. J. (2009). Submicroscopic infection in *Plasmodium falciparum*-endemic populations: a systematic review and meta-analysis. *The Journal of Infectious Diseases*.

[19] Mockenhaupt F. P., Rong B., Till H. (2000). Submicroscopic *Plasmodium falciparum* infections in pregnancy in Ghana. *Tropical Medicine & International Health*.

[20] Adegnika A. A., Verweij J. J., Agnandji S. T. (2006). Microscopic and sub-microscopic *Plasmodium falciparum* infection, but not inflammation caused by infection, is associated with low birth weight. *American Journal of Tropical Medicine and Hygiene*.

[21] McGready R., Davison B. B., Stepniewska K. (2004). The effects of *Plasmodium falciparum* and *P. vivax* infections on placental histopathology in an area of low malaria transmission. *American Journal of Tropical Medicine and Hygiene*.

[22] Okell L. C., Bousema T., Griffin J. T., Ouédraogo A. L., Ghani A. C., Drakeley C. J. (2012). Factors determining the occurrence of submicroscopic malaria infections and their relevance for control. *Nature Communications*.

[23] Mohammed A. H., Salih M. M., Elhassan E. M. (2013). Submicroscopic *Plasmodium falciparum* malaria and low birth weight in an area of unstable malaria transmission in Central Sudan. *Malaria Journal*.

[24] Falade C. O., Yusuf B. O., Fadero F. F., Mokuolu O. A., Hamer D. H., Salako L. A. (2007). Intermittent preventive treatment with sulphadoxine-pyrimethamine is effective in preventing maternal and placental malaria in Ibadan, south-western Nigeria. *Malaria Journal*.

[25] Hommerich L., von Oertzen C., Bedu-Addo G. (2007). Decline of placental malaria in southern Ghana after the implementation of intermittent preventive treatment in pregnancy. *Malaria Journal*.

[26] Luntamo M., Rantala A.-M., Meshnick S. R. (2012). The effect of monthly sulfadoxine-pyrimethamine, alone or with azithromycin, on PCR-diagnosed malaria at delivery: a randomized controlled trial. *PLoS ONE*.

[27] Menéndez C., Bardají A., Sigauque B. (2010). Malaria prevention with IPTp during pregnancy reduces neonatal mortality. *PLoS ONE*.

[28] Hill J., Hoyt J., van Eijk A. M. (2013). Factors affecting the delivery, access, and use of interventions to prevent malaria in pregnancy in sub-saharan africa: a systematic review and meta-analysis. *PLoS Medicine*.

[29] Thiam S., Kimotho V., Gatonga P. (2013). Why are IPTp coverage targets so elusive in sub-Saharan Africa? A systematic review of health system barriers. *Malaria Journal*.

